# Label-free detection of rare circulating tumor cells by image analysis and machine learning

**DOI:** 10.1038/s41598-020-69056-1

**Published:** 2020-07-22

**Authors:** Shen Wang, Yuyuan Zhou, Xiaochen Qin, Suresh Nair, Xiaolei Huang, Yaling Liu

**Affiliations:** 1 Department of Mechanical Engineering and Mechanics, Lehigh University, Bethlehem, PA 18015 USA; 2 Department of Bioengineering, Lehigh University, Bethlehem, PA 18015 USA; 3 Lehigh Valley Health Network, Lehigh Valley Cancer Institute, Allentown, PA 18103 USA; 4 College of Information Sciences and Technology and Huck Institutes of the Life Sciences, Pennsylvania State University, University Park, PA 16802 USA

**Keywords:** Renal cell carcinoma, Cancer imaging, Cancer screening

## Abstract

Detection and characterization of rare circulating tumor cells (CTCs) in patients' blood is important for the diagnosis and monitoring of cancer. The traditional way of counting CTCs via fluorescent images requires a series of tedious experimental procedures and often impacts the viability of cells. Here we present a method for label-free detection of CTCs from patient blood samples, by taking advantage of data analysis of bright field microscopy images. The approach uses the convolutional neural network, a powerful image classification and machine learning algorithm to perform label-free classification of cells detected in microscopic images of patient blood samples containing white blood cells and CTCs. It requires minimal data pre-processing and has an easy experimental setup. Through our experiments, we show that our method can achieve high accuracy on the identification of rare CTCs without the need for advanced devices or expert users, thus providing a faster and simpler way for counting and identifying CTCs. With more data becoming available in the future, the machine learning model can be further improved and can serve as an accurate and easy-to-use tool for CTC analysis.

## Introduction

Circulating tumor cells (CTCs) found in peripheral blood are originated from solid tumors. They are cells shed by a primary tumor into the vasculature, circulating through bloodstream of cancer patients, and colonizing at distant sites which may form metastatic tumors^1^. CTCs are an important biomarker for early tumor diagnosis and early evaluation of disease recurrence and metastatic spread in various types of cancer^2–6^. Early detection of CTCs provides high chances for patients to survive before severe cancer growth occurs^7^. The CTC count is also an important prognostic factor for patients with metastatic cancer^8–12^. For example, a study has shown that the number of CTCs is an independent predictor of survival in patients for breast cancer and prostate cancer^8–10^ , and the changes of the CTC count predict the survival in patients for lung cancer^12^.

However, the identification of the CTCs population is a challenging problem. Various approaches to identifying and isolating CTCs including antibody-based methods and physical-characteristics-based methods have been developed^13–19^. This task is difficult because of the low concentration of CTCs existing in a patient’s peripheral blood—a few CTCs out of 10 billion blood cells^20,21^, as well as heterogeneity in the characteristics of CTCs^22,23^. For example, the mechanism of CTCs maintaining metastatic potential during circulating is not well understood^24^; CTCs derived from some patients allow a cell line to be established, but CTCs from some others lose the capability of proliferation after a few hours of blood drawing^13^. Therefore, the incapability to draw a large volume of blood from patients leads to the need for improvements of CTC isolation methods so that CTCs can be detected in small sample volumes. Further, the inconsistency in the viability of CTCs hinders further explorations of relationships between the mechanism of patient-derived CTCs and tumor dormancy.

Locating specific target cells such as CTCs often requires tedious procedures. During the processes, CTCs need to be distinct from a huge amount of leukocytes via immunofluorescent labeling and fluorescent microscopy^25^, and identifying the CTCs via the fluorescent labeling images could be achieved with high-throughput^26,27^. Epithelial markers such as cytokeratin (CK), and epithelial cell adhesion molecules (EpCAM), are useful for detecting CTCs in patients. For example, CellSearch (Menarini Silicon Biosystems), an FDA-approved platform for CTC identification, is based on the overexpression of CK and EpCAM. However, detection of some types of CTCs such as CTCs in renal cell carcinoma (RCC) is limited by the lack of epithelial differentiation^28,29^. RCC shows low expression of epithelial markers, so such type of CTCs cannot be captured by labeling methods. In addition, fluorescent labeling comes with a few disadvantages. For example, as we already mentioned, most fluorescence imaging needs antibody-based fluorescence probes, which relies on overexpression of certain proteins on cell membranes, and such overexpression is usually not stable and largely relies on cancer type and patient^30^; photobleaching and phototoxicity occur in a short time after exposure under a fluorescent light source, and choosing proper fluorophores needs expert experience^31^; fluorescence staining often influences the viability of cells, thus impacts further culturing and analysis^32,33^. In contrast, intelligent cell identification and classification from low-resolution microscopic images allows a fast, cheap, and repeatable process. Thus, in this work, we aim to develop an automatic tool for accurate detection of CTCs as a promising step for diagnosis and clinical management of cancer patients.

Machine learning (ML) has become a superior tool for developing automated processes of classification, sorting, and detection^34–37^. ML algorithms build a mathematical or statistical model based on sample “training data” with known “ground truth” annotations, to make inference or predictions. There are traditional machine learning models such as random forest that can perform classification or prediction given high-quality features^38^, and deep learning models such as convolutional neural networks (CNNs) that can learn to extract features in an automatic fashion. For instance, CNN has been applied to the categorization of cell lines^36^ and red blood cells^37^. Chen et al*.*^39^ have integrated feature extraction and deep learning with high-throughput quantitative imaging enabled by photonic time stretch, achieving high accuracy in label-free cell classifications on selected white blood cells (WBCs) and cancer cells. They developed specialized hardware for the task of locating static, captured cells on a slide or in a device via a high speed, low resolution scan. However, instead of a simple and cheap experimental setup, their image acquisition is based on a time-stretch quantitative phase imaging system, and the representations of results can be improved by using samples from patients’ blood.

Our work is aimed at developing a fast and accurate technique for locating and counting tumor cells among the mixed cells after enrichment from whole blood. For state-of-the-art works using deep learning methods^36,37^, the model accuracy can be 85–95%, given a large amount of data with distinct differences between the candidate images. In this study, because the CTCs are rare cells from patients’ blood samples, the data size is relatively small. In the results and discussion section, we will discuss how we preprocess the raw dataset to mitigate challenges posed by the data size limitation. In this work, the CTCs and WBCs will be identified directly in regular bright field microscopic images without the need for advanced devices or time-consuming biological operations. The task will be achieved in a label-free fashion by using a CNN to classify cells detected in images of patient blood samples containing WBCs and CTCs.

## Materials and methods

The study was approved by the institutional review board of Lehigh Valley Hospital registered under FWA00000624 and all methods were carried out in accordance with relevant guidelines and regulations.

**Figure 1:**
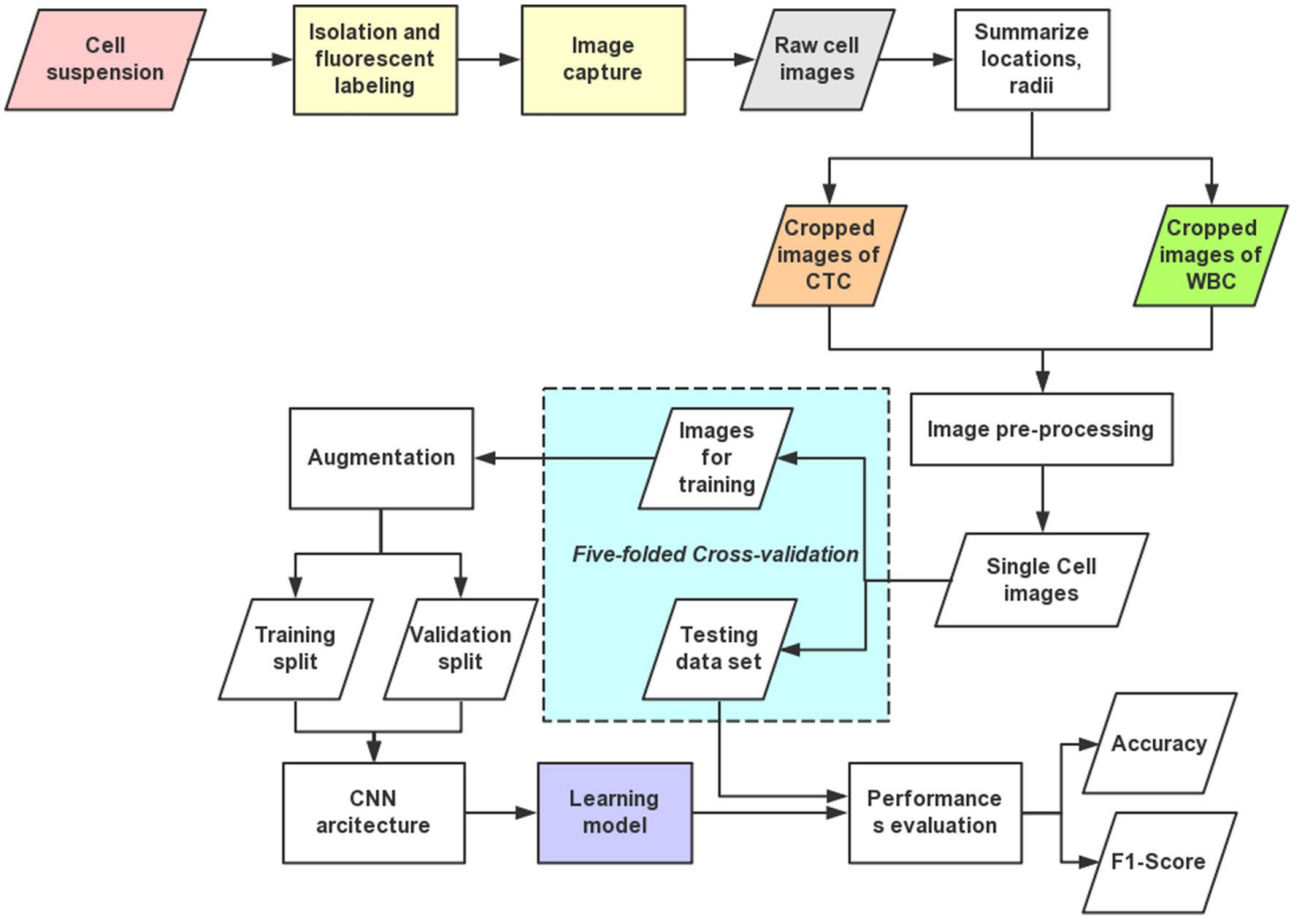
Flowchart of our deep-learning based analysis framework for microscopy images from isolated blood samples. Work steps include data preparation, image pre-processing, ML, and testing. The images collected from bright field and fluorescent microscopy are processed and cropped into images containing a single cell, which are then used as the training and testing raw data for the machine learning model with a deep CNN architecture.

Our work has the following steps: isolation and labeling the blood samples, image data collection, image processing, and training and evaluating our deep learning model. A flowchart shown in Fig. 1 demonstrates the work after acquiring the isolated and labeled blood samples.

### Blood samples

The peripheral blood samples from metastatic RCC (mRCC) patients were provided by Lehigh Valley Health Network, and healthy donor whole blood samples were provided by the University of Maryland. The sample collection processes for both have followed approved institutional review board protocols with patients providing informed consent.

The patient's whole blood was drawn in the 8.5 mL heparin tube and processed right away. 2 mL of whole blood was used for each batch of enrichment with EasySep Direct Human CTC Enrichment Kit. The human colorectal cancer cell line (HCT-116, *American Type Culture Collection (ATCC), USA*) and healthy donor whole blood were used in this work. WBCs used for the experiments were obtained from whole blood with red blood cell (RBC) lysis. In this experiment, 1 mL whole blood was lysed with 18 mL of RBC lysing buffer (*ThermoFisher*), followed by a 30-min incubation in the dark at room temperature. The mixture was then centrifuged at 500 g for 5 min. The supernatant was discarded, and the pellet was washed for 2–3 times with PBS. HCT 116 cells were pre-stained with *CellTracker (Thermo, USA) Red* and all WBCs were pre-stained with *CellTracker Green* prior use.

CTCs were isolated from peripheral blood samples of metastatic renal cell carcinoma patients. The isolated cells enriched from 2 mL of whole blood were triple washed using 1 × PBS (pH 7.4, Thermo). The enriched cells were mixed with 5 µL anti-hCarbonic Anhydrase 1 × and 2 µL Calcein AM*(BD Biosciences, USA)* to the cells and brought the final volume to 200 µL with PBS in a 1.5 mL sterile Eppendorf tube for staining. We used an efficient CTC immunomagnetic separation method (*EasySep* direct human CTC enrichment kit, Catalog #19657) with negative selection. We followed a manual EasySep protocol where peripheral blood was mixed directly with antibody cocktails (CD2, CD14, CD16, CD19, CD45, CD61, CD66b, and Glycophorin A) that recognize hematopoietic cells and platelets. The antibodies were labeled with unwanted cells, then labeled with magnetic beads and separated by EasySep magnet. The target CTCs will be collected from flow through and available for downstream analysis immediately. Live cells could be identified by being stained with *Calcein AM*, and CTCs isolated from renal cell carcinoma patients were stained with human carbonic anhydrase IX *(Abcam, USA)* PE-conjugated antibody. A live cell stained with the carbonic anhydrase IX PE-conjugated antibody would be finally identified as a CTC.

### Data collection

**Figure 2:**
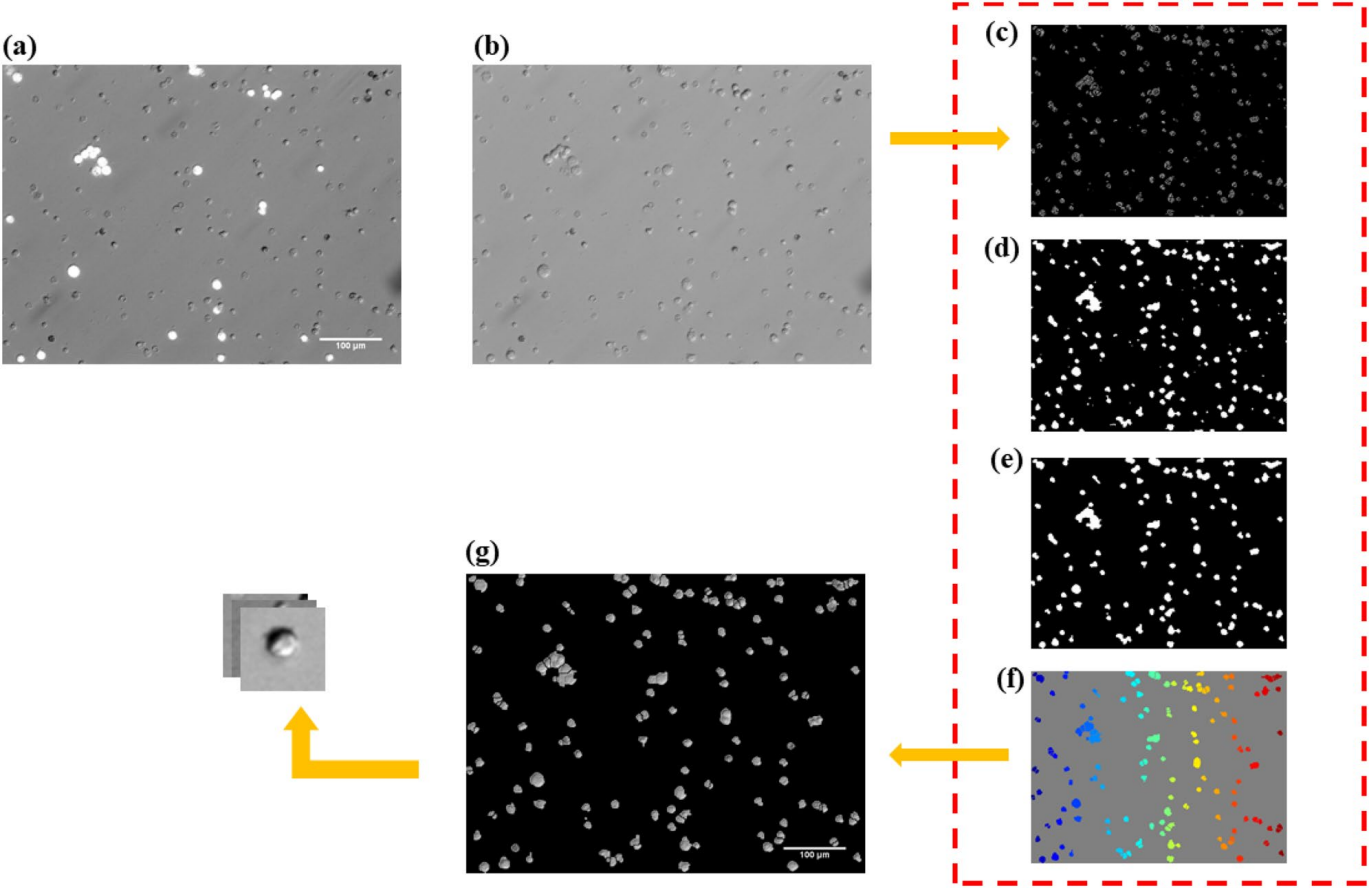
Demonstration of Image pre-processing on the raw image data with higher density of cell. (**a**) and (**b**) are the fluorescent labeled image and the corresponding bright field image, respectively; the bright field image is then processed in the toolbox (the dashed-line rectangle region), majorly including: (**c**) filtering by edge detection based on Otsu’s method, (**d**) flood-fill operation on the filtered image, (**e**) morphological opening operation that locates all cells and removes all irrelevant spots, (**f**) watershed transformation for segmentation. Each individual cell is visualized with a distinct color in this figure. (**g**) The appearance of segmented cells in the original bright field image. Then the bright field image can be cropped into individual cell images.

Optical images were obtained from fluorescent microscopy. Both immunocytochemically stained and bright field images were taken from tumor cell line mixed with WBC from healthy donor whole blood and the negative depletion of peripheral blood from renal cell carcinoma patients. The raw-cell microscopy images are acquired under an Olympus IX70-microscope, 640/480 microscopy bright field camera, with 20-X and 10-X scope magnification. The corresponding label images (Fig. 2a) for a subset of the raw-cell images (Fig. 2b) act as ground truth.

High resolution and high magnification images contain more details but acquiring them increases the total number of images to be captured and processed. Therefore, the selection of magnifier of the scope can be considered as a trade-off scenario. We chose 20-X as the magnification scope since it provides a reasonable image resolution for each cell (500 pixels), with acceptable number of images to acquire per testing sample.

### Image pre-processing

After raw images of cultured cells have been acquired, the first step of image pre-processing is applying Otsu’s filtering^40^ algorithm on the raw images to automatically segment the cells. Coelho *et al.*^41^ presented a discussion on the segmentation of this type of nuclear images. They evaluated different thresholding algorithms and found that Otsu’s filtering might behave poorly where extreme brightness from some very bright cells leads the algorithm to set a threshold between the very bright cells and the rest, instead of setting it between cells and background. The issue can be resolved by a manual adjustment of brightness, setting a maximum in brightness by judging whether the brightness would result in missing of cells with normal brightness, before the raw images are input into a segmentation toolbox we created. The toolbox is written in MATLAB and is available to be used for other segmentation purposes for bright field and fluorescent images.

On the toolbox, a raw image is processed through the Otsu’s filtering edge detection (Fig. 2c), flood-fill operation (Fig. 2d), and morphological opening operation (Fig. 2e), so that a watershed segmentation can be achieved (Fig. 2f). As one can see from the final segmentation result (Fig. 2g), although all cells contained in the image are located, there are irrelevant spots that have been mistakenly segmented as well. Therefore, after running a cropping algorithm to crop the segmented regions, it is not guaranteed that every cropped region corresponds to a single cell. To ensure the quality of training data for the CNN machine learning model, we manually select only the single-cell images from all cropped images and only use them to train the ML model. The label for a selected single cell image (WBC or CTC) is easily obtained from the label of the cell in the corresponding fluorescent image.

**Figure 3:**
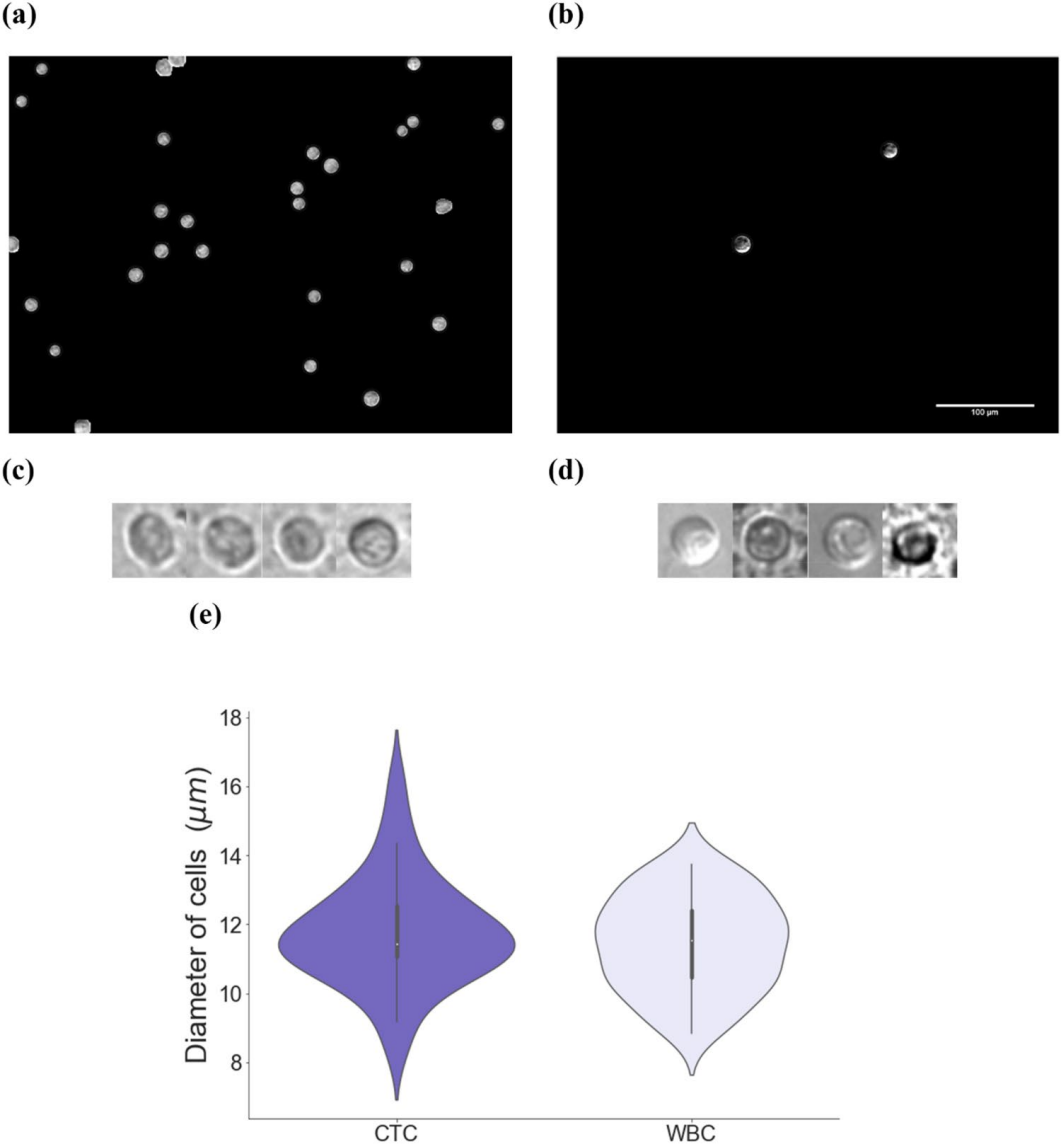
Demonstration of image data from patient blood. Selected images captured by the microscope from isolated patient blood samples are shown in (**a**), (**b**), the processed WBCs image and (**b**) CTCs image and cropped single (**c**) WBC and (**d**) CTC, respectively; (**e**) a summary of the size distributions of CTC and WBC cells: the average diameters of CTCs and WBCs are approximately both 11.5 µm, while the CTC has distinguishable wider range of size distribution.

An example of selected patient blood sample images captured from the microscope is shown in Fig. 3a (WBCs) and Fig. 3b (isolated CTCs). Some examples of cropped single-cell images are shown in Fig. 3c and d. The width and height of the cropped images are both 30 pixels. Because a cell may stay near the edge of a cell culture well where there are low intensity and cloudy background, a brightness and background normalization operation has been applied to all the cropped single cells. The cropped and normalized single-cell images are then used as the dataset for training and testing our ML model. The size ranges of CTCs and WBCs in patient blood samples have been collected and are shown in Fig. 3e. One can observe that both types of cells are similar in size, thus the size alone cannot be used to distinguish the two types of cells.

### t-SNE and the CNN model

Before performing training experiments, we generate the t-distributed stochastic neighbor embedding (t-SNE)^42^ plot for the training dataset to show the overall distribution of the data. t-SNE is a non-linear dimensionality reduction technique to generate a low-dimensional map from high-dimensional space, which finds the patterns in the data based on the similarity of input data points. The results of t-SNE can vary strongly due to the selection of the parameters of the algorithm. We use *scikit-learn*^43^ machine learning package to perform the t-SNE with the perplexity of 50 and the learning rate of 100. Under the correct setting of parameters, two distinct clusters corresponding to two different cell categories are clearly distinguished, which indicates that the deep learning network can extract the high-dimensional features and perform classifications on the dataset.

**Figure 4:**
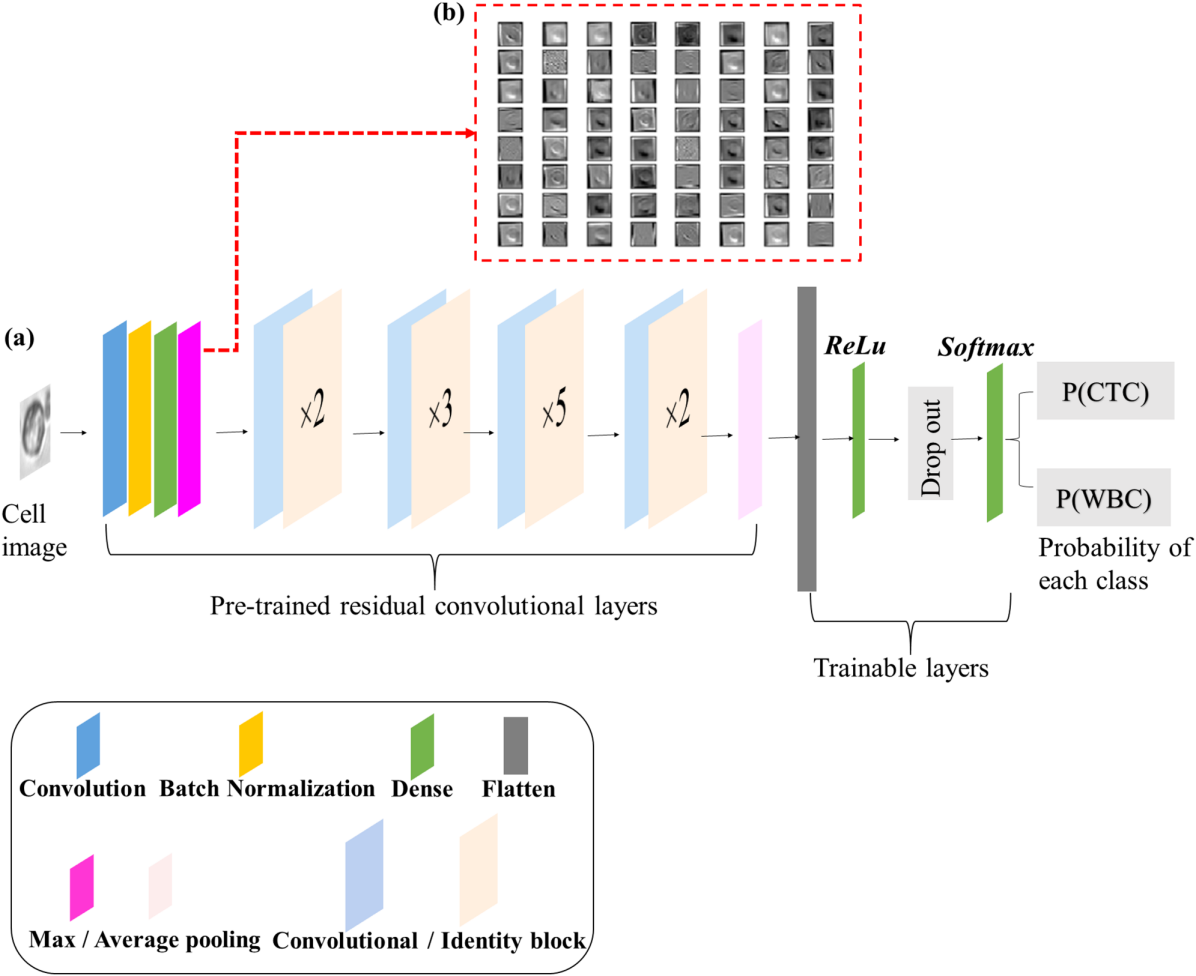
The architecture of the deep convolutional network, *ResNet-50*, for transfer learning and CTC–WBC cell classification (**a**), and the demonstration of features extracted (**b**) by the first convolutional block. The network receives the input data of cell images and predicts the probability of both classes. The network consists of five stages each containing convolution and identity blocks, and each of the blocks has three convolutional layers. The features of a cell image are extracted by the pre-trained convolutional layers.

The architecture of the machine learning model (Fig. 4a) is ResNet-50^44^, with input images of size 34 × 34 (resized from the cropped cell images), and binary categorical output. The convolutional layers are initialized with pre-trained weights learned from the ImageNet datasets^45^, a method that allows faster training and reduces the requirement of training data. These pre-trained weights are used for feature extraction, where the extracted features by the convolutional layers usually encode multi-scale appearance and shape information. For example, the first convolutional block directly takes the image data as input, extracts features and provides a feature map (Fig. 4b). Further feature extraction is applied by taking the feature map of the previous convolutional block as input for the next block. After the feature extractions, the pre-trained ResNet-50 is followed by trainable layers that contain a fully connected layer with *ReLu* activation function, a dropout layer with a dropout rate of 0.6, and a *softmax* activation function with a cross-entropy loss implemented to generate the predicted results. The model uses a learning rate of 0.0001 and is optimized by the *Adam* optimizer. The trainings are processed in mini-batch, with the batch size of 16.

## Results and discussion

As the comparison group, the training on cultured cell lines is based on 1745 single-cell images (436 cultured cells, 1309 WBCs). A total number of 120 cells (31 cultured cells and 89 WBCs) are tested. The combined performance has shown that all WBCs have been classified correctly, while 3 out of 31 cultured are misclassified as WBCs. The overall accuracy of this learning model is 97.5%. The training on patient blood samples is based on 95 single-cell images as raw input. The cell images originally came from two patients: 15 CTCs from one and 17 CTCs from the other. We have enhanced the training data before processing the training by applying data augmentations on the original dataset. The data augmentation increases the diversity of the original dataset. The most popular way to practice data augmentation is by creating a selected amount of new images by performing traditional affine and elastic transformations. The data augmentation provides a larger dataset, which helps improve the overall robustness of our WBC–CTC classification CNN model without additional laboring for the preparation of fluorescent labels. The expanded dataset includes single-cell images with different types of geometric transformations: rotation, shear transformation, horizontal and vertical reflection. Our augmented training dataset for each training experiment contains 1000 CTCs and 1000 WBCs.

**Figure 5:**
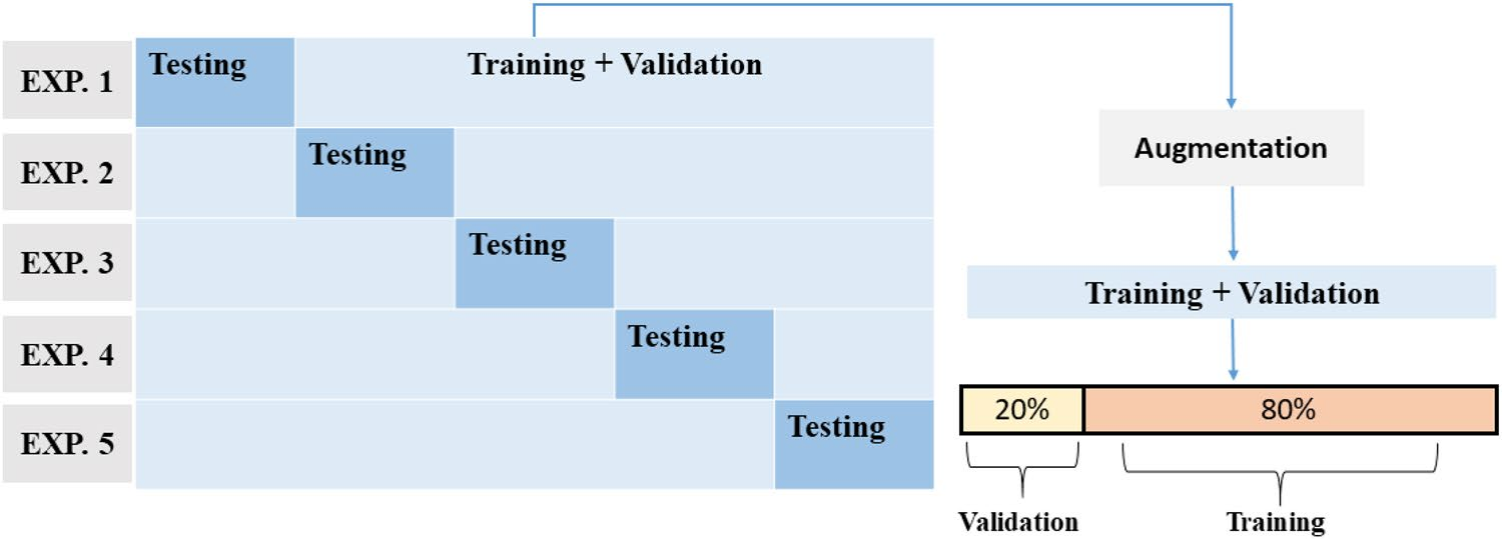
Five-fold cross-validation during training and testing experiments. The original data of single-cell images is shuffled and divided into five non-overlapped subsamples with equal number of images. One subsample is treated as the testing set in an experiment, and training is performed on the remainder of the dataset. The experiment repeats with each of the five subsamples once tested. In each experiment, the data for the training purpose is augmented and split into training (80%) and validation (20%) subsets, and then fits the model.

Due to the limited number of CTCs in patient blood samples, K-fold cross-validation^46^ is applied for measuring the overall performance. Cross-validation helps avoid performance differences in different runs of the learning algorithm, caused by the random split of training and testing data. We utilize five-fold cross-validation in our experiments. The original data is shuffled and divided into five groups with one group becoming the testing subset and the combination of the others becoming the dataset for training and validation. The training and validation data are then augmented for the training process. The final overall performance of the model is presented as the average of the five runs with different data as the testing set. More details on how we split the data and obtain training, validation, and testing datasets are described as seen in Fig. 5.

**Figure 6:**
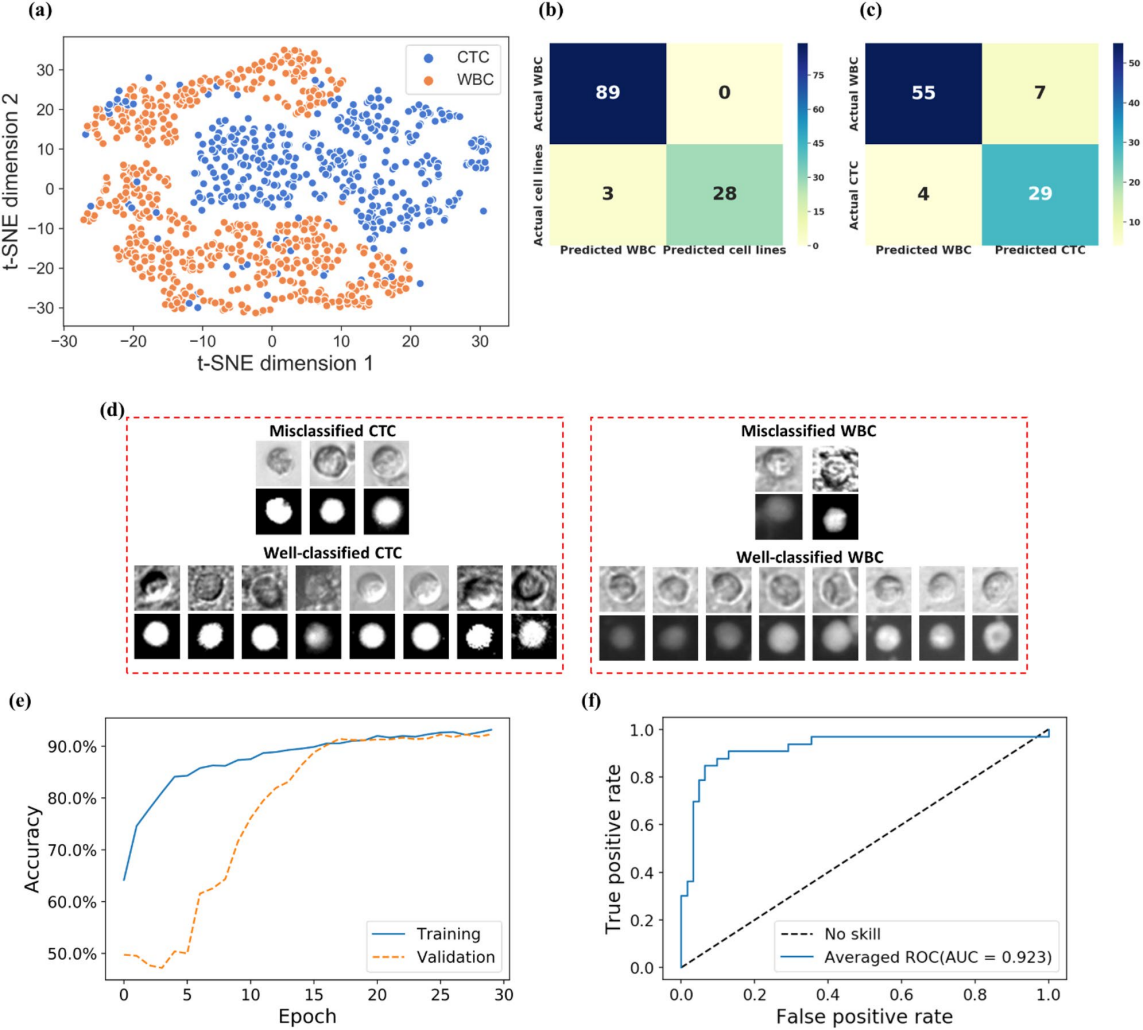
Trained model evaluation. (**a**) t-SNE plot of the training dataset showing the dimensionality reduction pre-processing for the training dataset. Confusion matrices for classification results of samples from (**b**) cultured samples, and (**c**) patient blood versus the WBCs. (**d**) Example misclassified and well-classified CTC images and WBC images. (**e**) The learning history of the training and validation at each epoch. (**f**) The overall ROC-AUC result for WBC and CTC classification by cross-validation. The ROC curve and AUC are the total/average performance of the five training experiments from the cross-validation process. As a comparison, a diagonal dashed line from the bottom left to the top right corners represent the non-discriminatory test.

After augmentation on the cell data, we visualize the training dataset by t-SNE algorithm. The t-SNE plot (Fig. 6a) shows the distribution of the first and second dimensions of the t-SNE map after performing non-linear dimensionality reduction by the algorithm on the training dataset. This t-SNE plot visualizes the high dimensional image data projected into a two-dimensional space, which helps to understand the overall distribution of the dataset. One can see from the output of t-SNE, samples from the two classes (CTCs and WBCs) form largely separate clusters in the two-dimensional space. We hypothesize that the separation of the two classes holds true in the high-dimensional space as well, and that explains why the trained deep learning model can reliably extract high-dimensional features and perform classification with high accuracy. The results of the deep learning model for cell image classification based on cultured cells and patient blood samples are summarized in Fig. 6b and c, respectively. Furthermore, examples of misclassified and well-classified CTCs and WBCs from the model are shown in Fig. 6d. We conjecture that the misclassifications could be due to noise or errors in the manual labeling process, and the inherent partial overlap between the distributions of the two classes (e.g. the CTCs mixed in the cluster of WBCs, and vice versa, as shown in the t-SNE plot). The averaged learning history from the five cross-validation experiments of the training and validation during epochs can be seen in Fig. 6e. The curves indicate that the model does not over-fit the problem and the network converges near the end of the training process. The testing results on cell images of patient blood samples show that the overall accuracy from the five-fold cross-validation is 88.4%, and the F-score, traditionally defined as the weighted harmonic mean of the precision and recall of the result, is 0.89. The F-score provides a measure of the overall performance of the model by considering the equal importance of precision and recall. As a comparison, in a recent study^47^, deep learning networks have shown the ability to unlock the hidden information in fluorescent images. The networks could classify fluorescent images of single cells including CTCs with a very high accuracy (96%). Although the bright field images of CTCs in our work have lower accuracy in classification due to the lack of fluorescent label information, our results that show nice convergence of the learning curve and promising accuracy with only limited amount of data demonstrate the potential of the proposed approach.

We also use the receiver operating characteristic (ROC) curve to show the performance of the model at all classification thresholds, and the corresponding area under the curve (AUC) value to indicate the performance of prediction on each experiment. Figure 6f shows the total ROC curve and the calculated averaged AUC, 0.923, for the classification of patient blood CTCs and WBCs. The high AUC indicates that the model has been successfully trained to distinguish CTCs from WBCs. The examples of misclassified and well-classified CTCs (Fig. 6d) show that the CTC images are either correctly detected or incorrectly classified as WBCs. Therefore, once a bright field image containing WBCs and CTCs are segmented and single-cell images are cropped, the trained model works as a binary classifier for the single-cell images without fluorescent labels. Note that the coordinates of all the cropped single cells in the bright field image are recorded during pre-processing. Therefore, after a predictive decision is made by the trained model, a label-free CTC count information for this bright field image can be generated when we combine the recorded coordinates and the corresponding predicted cell types. For further enumeration and characterization, this method can be combined with a sorting technique such as acoustic sorting^48^, where the upstream image machine learning results can be used to trigger pulse activation of acoustic forces that sort cells into different channels for isolation and characterization. Such combined label free image detection and label free sorting improves cell viability compared to a labelled approach and enables potential culturing of captured cells for personalized drug screening.

For future work, the CTC count information from RCC patients can also be combined with molecular characterization for clinical applications. Recent studies^29,49^ have shown that single-cell molecular characterization for CTC from RCC patients can unravel the information of clonal evolution. Characterization of CTCs that combines molecular characterization and statistical analysis by CellSearch during therapy can offer important information for the treatment selection for breast cancer patients^50^. As we mentioned, CTCs from RCC patient cannot be correctly isolated by CellSearch. If combined with molecular characterization, this label-free method of statistical analysis for CTCs would provide useful information to help choose the optimal therapy for mRCC patients.

## Conclusion

In this work, we have applied a deep convolutional neural network to classify cell images acquired from processed patient blood samples to detect rare CTC cells from mRCC patients. A software toolbox for pre-processing raw images acquired from the microscope is developed to apply Otsu's thresholding, segmentation, and cropping on the images. A manual selection process then ignores incorrect segmentations and chooses good single-cell images for training the CNN model. Ninety-five images containing single cells from patients are used as the original data, which is the source for training, validation, and testing datasets. Data augmentation is applied to expand the training and validation datasets. With the augmented data from the combination of two different patient blood samples and cultured cell images, the learning model yields 88.6% and 97% overall accuracy, on patient blood and cultured cells, respectively. The higher accuracy for the cultured cells indicates the potential of achieving a better learning model with more training images. We expect that the proposed method can work as an intelligent label-free detector for rare cells in isolated blood samples. More importantly, the proposed method is data-driven and can be further improved with more data samples.
